# Isolation and identification of marine microbial products

**DOI:** 10.1186/s43141-021-00259-3

**Published:** 2021-10-19

**Authors:** Sahar Saleh Mohamed, Sayeda Abdelrazek Abdelhamid, Radwa Hassaan Ali

**Affiliations:** 1 Microbial Biotechnology Department, Genetic Engineering Division, National Research Centre, Cairo, Egypt; 2 Masoud Hospital, Cairo, Egypt

**Keywords:** Bioactive compounds, Marine microbial, Natural products, MMNPs

## Abstract

**Background:**

The ocean is one of the world’s most important sources of bioactive chemicals in the marine environment. Microbiologists, ecologists, agronomists, taxonomists, and evolutionary biologists have been increasingly interested in marine microbial natural products (MMNPs) in recent decades.

**Main body:**

Diverse marine bacteria appear to get the ability to manufacture an astounding diversity of MMNPs with a wide range of biological actions, including anti-tumor, antimicrobial, and anti-cardiovascular agents according to numerous studies.

**Short conclusions:**

Innovative isolation and culture methodologies, tactics for identifying novel MMNPs via routine screens, metagenomics, genomics, combinatorial biosynthesis, and synthetic biology are all discussed in this review. There is also a discussion of potential issues and future directions for studying MMNPs.

## Background

The oceans, which cover more than 70% of the Earth’s surface, are not only rich in biodiversity but also a rich source of microorganisms with enormous potential. The maritime environment is home to a broad range of plants, animals, and microbes. Marine microbial communities, which include bacteria, viruses, and other microbes, have various advantages in biotechnological processes. Because almost all of those microbes can produce biochemical products, there is an ongoing need for new chemotherapeutics, particularly novel antibiotics, to address emerging illnesses and drug-resistant pathogens that are posing a serious threat to public health [1]. Over the last few decades, the discovery and development of novel medications from natural products (NPs) have played an important role. NPs are responsible for almost 28% of new chemical entities and 42% of anticancer medicines that have been launched to the market [2]. Microorganisms, in addition to plants and animals, are a valuable source of novel medicine development. More than 50,000 microbial natural products (MNPs) have been obtained, and they have played a key role in medicine development. The bulk of these have been identified from microorganisms that live on land [3]. Meanwhile, the emergence of severe antibiotic resistance in microbial pathogens including Gram-positive methicillin-resistant *Staphylococcus aureus* (MRSA) and vancomycin-resistant *S. aureus* (VRSA) is leading to an increase in the number of new diseases and pathogens. The current state of drug research highlights the importance of marine microorganisms as a source of novel secondary metabolites and the possibility to enhance the number of marine NPs in clinical trials [4]. The oceans, in contrast to the terrestrial environment, represent a vast and relatively untapped reservoir of new nanoparticles. Since the 1970s, over 15,000 structurally different NPs with a dizzying array of bioactivities have been discovered in marine habitats [5]. Researchers have been drawn to MMNPs because of their diversity in medication development. Over 30 chemicals produced from marine microorganisms, including didemnin B (AplidineTM) and thiocoraline, are being studied in clinical and preclinical trials for the treatment of various malignancies [6]. However, the search for MMNPs has only just begun [6, 7]. In this paper, we review the recent advances in MMNP discovery and development, especially addressing two important topics: (i) isolation and cultivation approaches of marine microorganisms and (ii) strategies for the discovery and development of MMNPs.

## Isolation and cultivation of marine microorganisms

The discovery of MMNPs is mostly attributed to marine microbes. Sixteen of the twenty marine anticancer drugs in clinical trials are generated from bacteria [8]. As a result, isolating and cultivating a new marine microbe could be a quick way to find new MMNPs. Microscopy and the fact that overall cell counts are typically three orders of magnitude larger than the number of colony-forming units show this [9]. The majority of microorganisms from the environment (> 99.9%) do not form colonies on the nutrient-rich agar medium that has been utilized to isolate marine bacteria in the past. This limited culture ability could be attributed to the artificial conditions seen in most culture media, such as the lack of essential nutrients needed for growth [10]. Despite the availability of a variety of molecular methods for analyzing microbial communities, cultivation-based analyses are far from redundant because only the isolation of individual bacterial species in pure culture allows for a comprehensive characterization of physiological properties and a full assessment of application potential (e.g., bioactive compounds) [11]. As a result, developing isolation and cultivation methods is a precondition for studying marine microorganisms in depth.

### Isolation of diverse marine microorganism using pretreatment strategies

Specific groups of marine microorganisms, particularly the less prevalent bacteria, can be isolated using pretreatment procedures. To favor the isolation of specific genera and to improve the recovery of these microorganisms, a variety of pretreatment methods are used including enrichment chemical and physical techniques such as exposure to phenol, dry heat, sucrose-gradient centrifugation, and filtration through cellulose membrane filters [12–16]. These pretreatments reduce or eliminate the possibility of contamination, making it easier to isolate slow-growing marine bacteria. Yamamura et al. [12] established a sucrose centrifugation method for the comparatively high specific isolation of *Nocardia*, a less abundant Actinomycete, to separate it from other Actinomycete by centrifugation. *Nocardia* cells were enriched in the 20% sucrose layer by contrast, larger numbers of *Streptomyces* spp. were found in the range of 30-50% sucrose layers and *Micromonospora* was observed in the range 20-30% sucrose layers but in relatively low numbers, also *Actinoplanes* was only recovered from the 10% sucrose layer. Bredholdt et al. [13] investigated the variety of actinomycetes in the marine sediments of the Trondheim Fjord, Norway, using various pretreatment techniques such as super high-frequency radiation, UV irradiation, extremely high-frequency radiation, and cold shock. In addition to the predominant genera *Micromonospora*, and *Streptomyces* representatives of *Actinocorallia*, *Knoellia*, *Glycomyces*, Actinomadura, *Nocardia*, *Nonomuraea*, Nocardiopsis, *Rhodococcus*, *Pseudonocardia*, *and Streptosporangium* genera, were isolated from marine. Jensen et al. [14] used 8 selective isolation techniques include dry/scrape, dry/stamp, dilute/heat, dry/dilute, dilute/heat + dry/stamp, and freeze/dilute to isolate actinomycete from 275 marine samples collected around the Guam island. The most common actinomycetes were discovered, including the seawater-dependent “*Salinospora*,” a new genus of the Micromonosporaceae family. Furthermore, members of two important novel clades linked to Streptomyces spp., MAR2, and MAR3, were grown and suggested to represent new species within the *Streptomycetaceae* that can be easily cultured using low-nutrient media. Kjer et al. [16] explained in detail the isolation and cultivation procedures for fungi associated with algae, sponges, and mangroves. Marine-derived fungi can also produce a slew of new bioactive secondary metabolites that could be used as innovative therapeutics or plant protection agents.

### Selection and design of culture media for the biodiversity of marine microbes

**Fig. 1:**
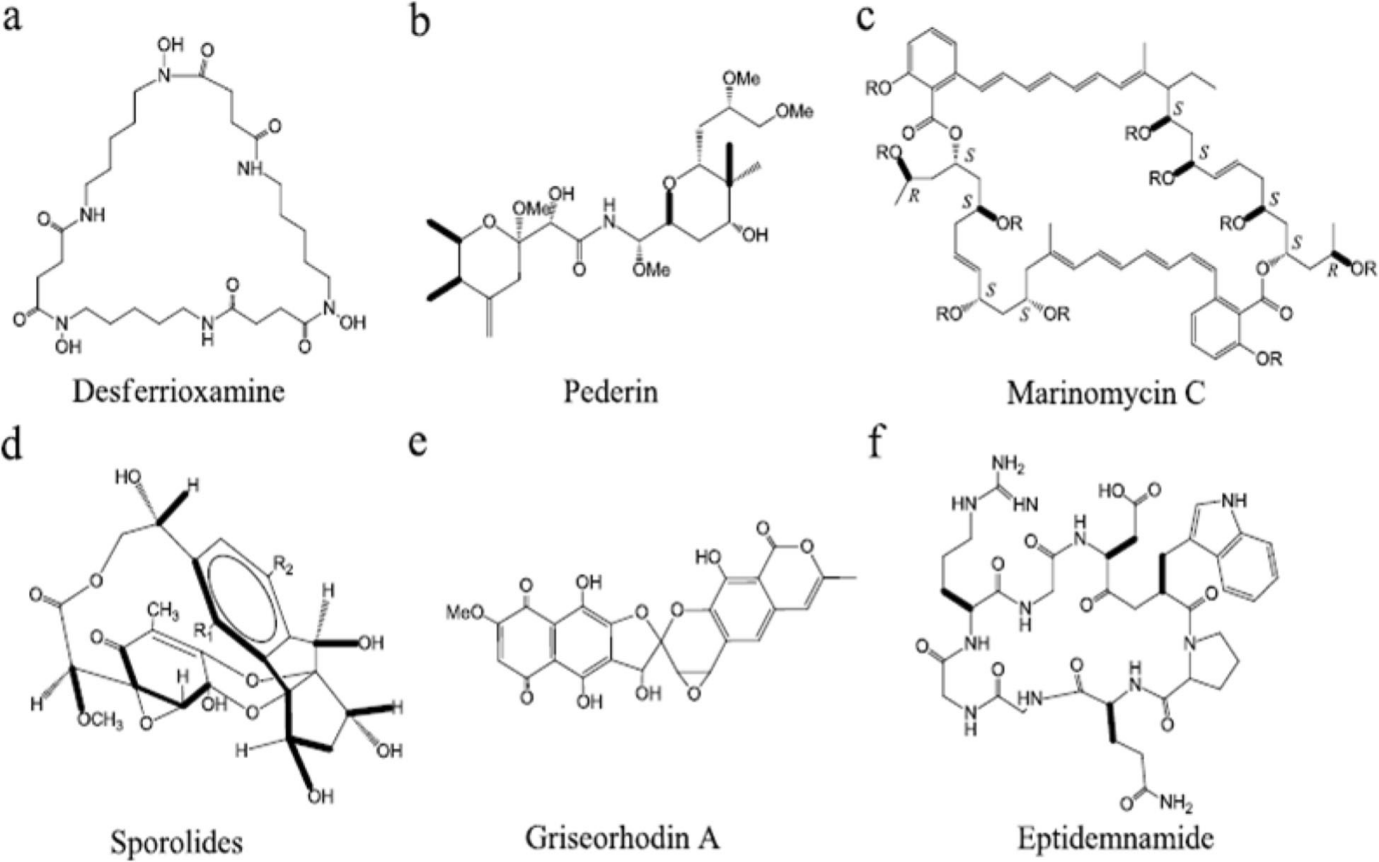
Structures of some marine microbial bioactive metabolites

Nutrients, energy sources, and proper physicochemical conditions are necessary for growth of microorganism. Various bacteria require specific nutrients in specific amounts and types. Most marine microorganisms have specific nutritional (sponge extract [17]) or chemical needs for development (siderophores [18], non-traditional electron donors, electron acceptors, and signal molecules [19]). Bruns et al. [20] employed artificial brackish water with different carbon substrates such as starch, agarose, laminarin, chitin, xylan, and glucose at low concentrations (200 μM each) as the growth medium to improve the cultivation efficiency of bacteria from the Gotland Deep in the central Baltic Sea. In compared to prior research [21], this approach generated much higher culture efficiency in fluid media, up to 11%. Furthermore, at a low concentration of 10 μM, adding cyclic AMP (cAMP), N-oxohexanoyl-DL-homoserine lactone, or N-butyryl homoserine lactone can greatly improve culture performance. The most efficient inducer was cAMP, which resulted in cultivation efficiencies of up to 100% of total counts of bacteria. Other culture conditions such as medium ionic strength are important to growth of marine microbes. Genus of marine actinomycetes desferrioxamine (Fig. 1a), arenamides, saliniketals, salinosporamide, and arenimycin are examples of beneficial secondary metabolites produced by *Salinispora* [22–26]. Three species of *Salinispora*, *S. tropica*, *S. arenicola*, and *S. pacifica*, require a high ionic strength for growth, according to Tsueng et al. [27]. *Salinispora* has a growth requirement for divalent ions calcium and magnesium, as well as a growth requirement for ionic strength (8.29 to 15.2 mS/cm) in both lithium chloride-based and sodium chloride-based environments. *S. arenicola* has a lower ionic strength growth requirement than *S. tropica* and *S. pacifica*. They also developed a potassium chloride-based salt formulation containing low sodium concentration (5.0 mM) to support the growth of *S. tropica* NPS21184 and its production of salinosporamide A (NPI-0052). Although *S. tropica* does not have a seawater growth requirement, it requires a specific combination of salts to provide a balance of salts and maintains a high enough ionic strength for growth [28].

### Innovative cultivation approaches to recover less-cultivable or unculturable marine microbes

Only around 1% of bacteria are capable of being cultured, and these are the ones that are not [29], thus finding means to cultivate the uncultured bulk of the microbial world for the discovery of MMNPs is a top priority for microbiologists today. The antitumor chemicals pederin (Fig. 1b), mycalamide A, and onnamide A were found by an uncultivated bacterial symbiont of the marine sponge *Theonella swinhoei* [30]. Several researches in recent years have shown that by refining traditional methods, several “not-yet-cultured” species may be produced. Slow-growing microbes thrive in nutrient-poor environments and may be inhibited by substrate-rich conventional media [11]. Nutrient-rich culture media may favor the growth of faster-growing microbes at the expense of slow-growing species that always thrive in nutrient-poor environments and may be inhibited by substrate-rich conventional media. As a result, recovering uncultivated microorganisms requires a culture that mimics the natural environment. For example, hitherto uncultivated microorganisms, such as the ubiquitous SAR11 marine bacterio-plankton clade, have been cultured in saltwater [31, 32]. Furthermore, hitherto uncultivable microorganisms from various marine habitats have been successfully cultivated using dilute nutrient media techniques [10, 32, 33]. At in situ substrate concentrations, Stephanie et al. [33] identified and grew four new cell lineages belonging to hitherto uncultured or described marine *Proteobacteria* clades (three orders of magnitude less than common laboratory media). The clades OM43 (subclass), SAR11 (subclass), SAR92 (subclass), and OM60/OM241 (subclass) were shown to be connected to these four distinct cell lineages. Furthermore, under laboratory circumstances, certain germs can only be successfully cultured in the presence of other microorganisms. Growth-stimulating chemicals generated by helper microorganisms are frequently used by unculturable strains. As a result, uncultivable species were frequently given culture supernatants or cell-free extracts from helper strains as growth stimulants [34–37]. In the marine environment, many microorganisms grow slowly; consequently, extended incubation time is required for the cultivation of such microbes at low substrate concentrations in specified media, with the added benefit of minimizing bacterial competition among mixed populations [11]. To separate the primary bacterioplankton lineages in the East Sea, Western Pacific Ocean, Song et al. [32] used modified dilution-to-extinction cultivation with prolonged incubation at low temperature. Extinction cultures of the *Roseobacter*, *SAR11*, *OM43*, and *SAR92* clades were isolated after 20 and 24 weeks of plate incubation. Three reviews [11, 29, 38] have explored the potential causes of unculturability as well as various different ways for cultivating unculturable bacteria despite the fact that many “not-yet-cultured” species can be cultivated, and the molecular processes of unculturability have been discovered, finding new cultivation tactics is still an effective step for all microbiologists and plays a significant role in identifying new species. Various techniques such as high throughput screening (HTS), diffusion chamber system [37, 39], encapsulation method [34, 40], soil substrate membrane system [41–44], filtration method [45], density-gradient centrifugation, extinction dilution [46], and fluorescence-activated cell sorting (FACS) [46] have had a significant impact on recovering as-yet-uncultivated species. Zengler et al. [10] used flow cytometry to detect microdroplets harboring microcolonies after encapsulating cells in gel microdroplets for massively parallel microbial culture under low nutrient flux circumstances. Kaeberlein et al. [39] created a diffusion chamber that allowed substances from the natural environment to pass across a membrane, allowing hitherto uncultivated bacteria to be separated from sea sediment. They discovered that these isolates only established colonies on artificial media when other bacteria were present. Diffusion chambers of this type have also been used in the study of seldom cultivated microorganisms from the sea [37]. The substrate membrane utilized for micro colony development of uncultivated bacteria systems [42] is one of the most recently created, creative techniques. A polycarbonate membrane support and soil extract as a substrate are used in this system. It enables the microculture of novel bacterial strains, the detection of living micro colonies using viability staining, and the micromanipulation of colony separation [41]. Overall, new methodologies and techniques for isolation and cultivation will be required to recover more cultivated and as-yet-uncultivated microbial species in order to uncover structurally distinct chemicals with interesting biological activity.

## Discovery and development of MMNPs

The isolation of new marine microorganisms, as well as the innovation of screening methodologies, encourages the identification and development of novel MMNPs. In this part, five screening methodologies for MMNP discovery and development are discussed: (i) conventional screening, (ii) metagenomics, (iii) genomics, (iv) combinatorial biosynthesis, and (v) synthetic biology. Table 1 provides an overview of the important studies of MMNPs identified using various approaches.

**Table 1:** Representative examples of MMNPs discovered by various methods

Compounds	Host	Method	Activity	Reference
marinomycin	*Marinispora* sp. CNQ-140	bioactivity-guided screening	antitumor	[47]
medermycin	*Streptomyces* sp. 16	gene-guide screening	antimicrobial and antitumor	[48]
pederin	uncultured *Pseudomonas* sp.	metagenomics	antitumor	[30]
salinilactam A	*Salinospora tropica*	genomics	antitumor	[25]
salinosporamide XI/X2	*Salinospora tropica*	combinatorial biosynthesis	proteasome inhibitor	[49]
eptidemnamide	*Prochloron* spp.	synthetic biology	antitumor	[50]

### MMNPs discovery via conventional screenings

**Fig. 2:**
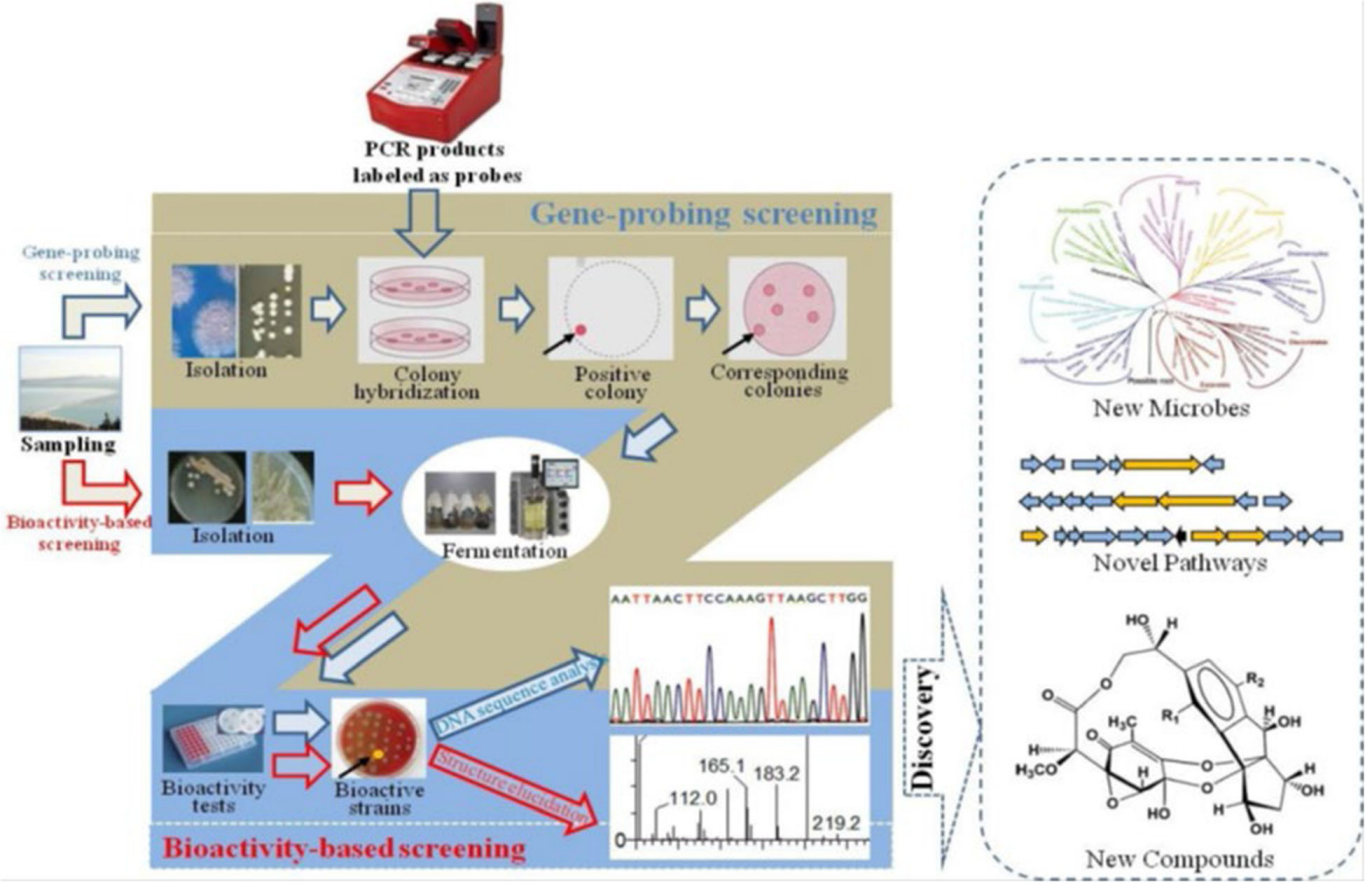
The combined strategy of gene-based screening and bioactivity-based screening for marine microbial natural products’ (MMNPs) discovery

Bioactivity-guided screening and gene-guided screening are two of the most common screening approaches. Using the culture supernatant or extract of cell pellet, bioactivity-guided screening can discover activity such as antiparasitic, anticancer, antibiotic, antiviral. Marinomycins A–D (Fig. 1c), for example, were isolated from the saline culture of *Marinispora* sp. CNQ-140 based on significant antibacterial activities (the values of MIC are 0.1–0.6 μM) against drug-resistant pathogens (MRSA) and impressive and selective cancer cell cytotoxicities (the MIC50 values are 0.2–2.7 μM) against six melanoma cell lines in the National Cancer Institute’s NCI-60 cell line panel [47]. Gene-guided screening has been used to look for target genes related with NPs biosynthesis pathways in order to find novel sources of bioactive secondary metabolites including the fragments between ketosynthase and methylmalonyl-CoA transferase of polyketides (PKS) type I [51], dTDP-glucose-4, 6-dehydratase (dTGD) gene [48], enediyne PKS ketosynthase gene [52], *O*-methyltransferase gene [53], P450 monooxygenase gene [54], polyether epoxidase gene [55], 3-hydroxyl-3-methylglutaryl coenzyme A reductase gene [56], and halogenase gene [57]. Gene-based screening, when paired with homology-based searches and phylogenetic analysis, has the highest promise for predicting the generation of novel secondary metabolites carried by isolates or environments. This predictive capability offers a quick and easy way to avoid isolating known compounds or finding strains that produce compounds in a certain structural class [58]. Chen et al. [48] used PCR to explore at the distribution of the dTGD gene and the diversity of putative 6-deoxyhexose (6DOH) glycosylated compounds in 91 marine sediment-derived bacteria from 48 OTUs and 25 species. The dTGD gene was found in 84% of the strains, indicating that the 6DOH biosynthesis pathway is ubiquitous in these marine sediment-derived bacteria. The BLASTp results also revealed that the possible 6DOH glycosylated molecules had a wide chemical diversity. The findings showed that phylogenetic analysis of the dTGD gene can be used to predict the structure of glycosylated compounds from freshly isolated strains, which can help with chemical purification and structure identification processes including medermycin and chromomycin A3 [48]. In the absence of fully built pathways or genome sequences, gene-guided screening gives a bioinformatics assessment of secondary metabolite biosynthesis potential. This simple and quick prediction, which includes new secondary metabolites or recognized chemicals in the strains, can help speed up the MMNP discovery process by allowing researchers to pick strains for fermentation and chemical investigation (Fig. 2). For the discovery of marine microbial natural products (MMNPs), a combination technique of gene-based screening and bioactivity-based screening was used. Due to the drawbacks of bioactivity/gene-guided screening, a combination technique of gene and bioactivity screening (Fig. 2) may be more effective in obtaining useful strains capable of synthesizing novel bioactive chemicals. For 109 bacteria isolated from four South China Sea sponges, Zhang et al. [59] used PCR to screen for nonribosomal peptide synthetase (NRPS) genes. Based on 16S rDNA sequences, fifteen bacteria were found to have NRPS genes and were divided into two phyla: *Firmicutes* (13 of 15) and *Proteobacteria* (2 of 15). Based on phylogenetic analysis of the conserved domain, the majority of the NRPS fragments (11 of 15) exhibited a 70% resemblance to their nearest relatives, indicating the uniqueness and diversity of these NRPS genes. The antimicrobial activities of all 15 bacteria with NRPS genes were found, with the majority of them having broad-spectrum activity against fungi and bacteria, indicating the chemical diversity of sponge-associated bacteria’s biologically active metabolites and the possible role of bacterial symbiosis in the host’s antimicrobial chemical defense [59]. In summary, the combined gene and bioactivity technique will be beneficial in the development of novel MMNPs.

### MMNPs discovery via metagenomics

More than 99% of bacteria are difficult to cultivate in the lab. The majority of bacteria must therefore be discovered using culture-independent approaches [60]. By extracting complete ambient DNA, metagenomics allows direct access to the genomes of whole environmental microbes. The introduction of eDNA into a suitable host and screening of these enormous eDNA libraries for bioactive clones is an effective technique to access NPs encoded by the genomes of previously uncultured microorganisms [61]. eDNA libraries derived from microbial populations in unusual or unknown conditions are thus significant resources for MMNP discovery [62, 63]. The finding of novel structures with varied bioactivities, including as violacein, terragines, turbomycins, and indirubin using the metagenomic technique has demonstrated that this technology is a viable option for NP drug development in uncultivable microorganisms [5]. The two primary methodologies for eDNA library screening are function-driven analysis (e.g., bioactivity assay) and sequence-driven analysis (e.g., DNA probe) [61, 62, 64]. The essential genetic components for the intact cluster assembly are identified through sequence-based screening using homologous PCR or clone hybridization [60]. Feng et al. [65] exploited transformation-associated recombination to reassemble two described eDNA clones (called AB185 and AB649) into a complete type II PKS biosynthetic pathway and heterologously expressed in *S. albus*, based on the sequence-driven analysis. C, F, G, and H, four new metabolites fluostatins, were discovered in assembled clones but not in AB649 cultures alone [65]. Furthermore, the creation of a variety of model microbial systems that can act as heterologous hosts for eDNA expression could broaden the NP repertoire. Craig et al. [66] investigated the β--Proteobacterium *Ralstonia metallidurans* as a potential model system for eDNA library expression. Cosmids conferring the production of unique pigmented/antibacterial compounds in *R. metallidurans* clones did not operate in *E. coli*, which was surprising. This innovative technique extracted novel chemicals with a simple biosynthetic scheme, such as patellamide, indigo, and pederin [30]. As a result, a wider range of model hosts will be used to expand the number of metabolites discovered in future metagenomic research. The inability to efficiently acquire complete gene fragments and incompatibility of expression elements such as the promoter in a heterologous host may be limitations of metagenomics. Although it is still too early to draw any conclusions about metagenomics-based MMNP discovery, we believe that rapid advances in synthetic biology, such as large DNA fragment assembly techniques for artificial genome synthesis and synthetic microbial chassis suitable for various classes of MMNP biosynthesis, will greatly aid active expression of the entire DNA cluster.

### Mining diverse biosynthetic clusters of MMNPs via genomics strategies

Because secondary metabolites like NRPS, PKS, and PKS–NRPS hybrids are commonly biosynthesized by large multifunctional synthases that progressively assemble tiny carboxylic acid and amino acid building blocks into their products, genomics has been utilized to MNP identification [25]. With advancements in DNA sequencing and bioinformatics, it is now possible to quickly identify the gene cluster of bioactive chemicals and predict their chemical structure in silico using genomic data. These structural predictions can help with compound purification, structure confirmation, and the identification of new chemical entities [67]. To date, the genome sequence tags (GSTs) probe has effectively discovered over 450 MNP gene clusters using the genome scanning technique [68]. This approach was utilized by Zazopoulos et al. [68] to isolate an enediyne (a potent family of antitumor antibiotics) gene cluster from a range of marine actinomycete strains. A conserved cassette of five gene clusters, including a novel family of PKS, was discovered in a comparative examination of five biosynthetic loci characteristic of enediynes. In all enediynes, the enediyne PKS plays a role in the production of the highly reactive chromophore ring (or “warhead”) structure. Actinomycetes have a high prevalence of the enediyne warhead cassette, according to genome scanning study. The number of gene clusters involved in NP production is far more than the number of metabolites identified in microorganisms through genome sequencing [69]. Actinomycetes’ genome study, for example, reveals a plethora of cryptic gene groups [70–72]. On average, each actinomycete strain appears to have the ability to create around 20 NPs, while standard screening regularly only detects about two NPs per strain [69]. The powerful proteasome inhibitor salinosporamide A [73], the unprecedented halogenated macrolides sporolides A and B (Fig. 1d) [74], lymphostin, and salinilactam are among the chemicals generated by *S. tropica.* By genome sequencing, Udwary et al. [25] identified all secondary metabolic biosynthetic gene clusters in *S. tropica* CNB-440. The majority of the 17 biosynthetic clusters have never been seen before. This strain has the most diversified polyketide biosynthetic pathways, as well as the greatest proportion of its genome (9.9%) dedicated to NP production. Not only has bioinformatic analysis benefited in the structure elucidation of the polyene macrolactam salinilactam A, but it has also aided in the genome assembly of the highly repetitive slm loci [25]. Winter et al. [75] outlined the findings in this field and assessed the field’s future prospects. More than 50 actinomycete genomes are being sequenced around the world [76, 77], making genomics more appealing. When a result, as more genome-mining techniques is developed, genomics will become more potent in the discovery of MMNPs.

### Diversified MMNPs via combinatorial biosynthesis

Chemical synthesis presents a problem when it comes to generating derivatives from NPs due to their complicated architectures. Combinatorial biosynthesis is a great approach for increasing the chemical variety of NPs to solve this difficulty. It entails manipulating the biosynthetic clusters of NPs genetically in order to obtain new/altered structures that would be difficult to synthesis using existing approaches. As a result, it is a valuable addition to typical microbial drug discovery strategies. MMNPs’ biosynthetic elements differ from those produced from terrestrial sources in that they contain halogenase and other new enzymes. Combinatorial biosynthesis can produce unnatural compounds by heterologous expression of biosynthetic genes from many sources [78]. In The chlorinase SalL halogenates S-adenosyl-l-methionine (SAM) in the biosynthesis of salinosporamide A to form 5′-chloro-5′-deoxyadenosine (5′-ClDA) in a nucleophilic substitution similar to that of the fluorinase in the fluoroacetate producer *S. cattleya* [79]. SalL’s substrates are also bromide and iodide, rather than fluoride. Eustaquio et al. [79] used synthetic 5′-fluoro-5′-deoxyadenosine (5′-FDA) to make fluorosalinosporamide, a novel salinosporamide derivative, in a salL-knockout mutant of *S. tropica* that was devoid of salinosporamide A. Moreover, a new shunt in the phenylalanine pathway to L-3-cyclohex-2′-enylalanine (CHA) residue in salinosporamide A (SalX disruption in *S. tropica* mutant) through combinatorial biosynthesis enabled the generation of not only antiprotealide but also other unnatural salinosporamide derivatives (salinosporamide X1 and salinosporamide X2) with C5 modifications in the salinosporamide family of potent proteasome inhibitors [49]. Despite the numerous triumphs of combinatorial biosynthesis [50, 80], there is a stumbling block in the form of the designed compound’s productivity being lower than that of the parent MMNPs. Furthermore, despite the wide variety of possible structural alterations, combinatorial biosynthesis does not yield compound libraries with great diversity [69]. As a result, these issues in combinatorial biosynthesis will most likely be solved within the next decade.

### Diversified MMNPs via synthetic biology

MMNPs accumulate at very low levels in native producers, despite the vast structural diversity in their libraries providing a pharmacological pool for public health. More MMNPs can be generated to enable structural elucidation, activity investigation, and potentially clinical trials, thanks to advancements in microbial cultivation and fermentation technology. Ordinary people, on the other hand, cannot afford the therapeutic cost of MMNPs due to their higher manufacture cost. In addition, a growing number of MMNPs found through genome or environmental DNA sequence mining are awaiting confirmation. With synthetic biology’s rapid advancement, it could be a potential technique for improving the production of recognized chemicals or activating silent gene clusters. Because of the rapid advancement of synthetic biology, it may be a potential technique for improving health. The development of genome-wide genetic manipulation techniques such as hierarchical conjugative assembly genome engineering (CAGE) [81] and multiplex automated genome engineering (MAGE) [82] is based on the development of genome-wide genetic manipulation techniques, and synthetic biology can create natural or artificial biosynthetic pathways in the host microorganisms for MMNPs using plentiful genetic resources (e.g., functional genes taken from many sources, controlled regulatory elements [74, 83], and synthetic RNA/protein scaffolds [75, 76]). Given the triumphs of synthetic biology, the high yields of erythromycin precursor 6-deoxy-erythronolide B, taxol precursor taxadiene, and artemisinin precursor amorphadiene in surrogate hosts [77, 78] are the finest examples, indicating its enormous potential for the creation of useful MMNPs. Compatibility between host microbes and synthetic materials of the intended product, including pathway gene expression, enzyme activity, and precursor supply, are essential concerns for host selection in synthetic biology, in addition to efficient DNA manipulation and transfer methods. Although the production of NPs is quite similar in terrestrial and marine microbes, developing marine-derived hosts (e.g., marine-derived cyanobacteria, actinomycetes, and fungi) will be useful in the quest for heterologous MMNP expression. Non-essential DNA sections have been removed from genome-minimized microorganisms [81], making them a viable biological chassis for MMNPs. All of the preceding research and methodologies are paving the way for the synthesis of MMNPs through heterologous expression. The use of heterologous production of full NP gene clusters to investigate the role of genes and gene clusters involved in metabolite manufacture is a clever method [79]. At Heron Island, Queensland, Australia, Piel et al. [78] isolated Streptomyces sp. JP95 from the marine ascidian Aplidium lenticulum. The telomerase inhibitor griseorhodin A (Fig. 1e), produced by *Streptomyces* sp. JP95, is the most heavily oxidized bacterial polyketide known, with a unique epoxyspiroketal moiety that is critical for its activity [78]. The griseorhodin biosynthetic cluster encodes an extraordinary number of functionally distinct oxidoreductases (encoded by 11 different ORFs), which are engaged in the oxidative modification of a polyaromatic tridecaketide precursor via three carbon–carbon bond cleavage. *Streptomyces* sp. JP95 is surprisingly resistant to foreign DNA introduction via a number of ways, preventing knockout tests to establish that the grh cluster is involved in griseorhodin production. Heterologous expression of the entire grh cluster in S*. lividans* on a suitable shuttle cosmid is an alternate method. As a result, the S. lividans ZX1 (pMP31a) modified strain can successfully manufacture griseorhodin A and three other similar chemicals. Another successful case is that a new cyclic peptide eptidemnamide (Fig. 1f) was produced by an engineered *E. coli* by replacing the ulithiacyclamide (5)-coding region from *patE2* (responsible for heterocyclization of cysteine, serine and threonine residues, and *N*-terminal to *C*-terminal cyclization to afford the final patellamides) with a wholly artificial construct and expression of the *pat* cluster (responsible for patellamide biosynthesis) from the obligate cyanobacterial symbionts *Prochloron* spp., thereby demonstrating for the first time that the whole biosynthetic cluster of MMNPs can be functionally expressed in the surrogate host [50]. Despite the fact that synthetic biology is a new science with much more work to be done on its theory and methodology, there is little question that it will usher in a new era of MMNP development [80].

## Conclusion

Marine microbial natural products (MMNPs) have gotten a lot of interest recently from a variety of sources. Diverse marine bacteria appear to be capable of producing a vast diversity of MMNPs with antimicrobial, anti-tumor, anti-inflammatory, and anti-cardiovascular properties. MMNPs include metagenomics, genomics, combinatorial biosynthesis, and synthetic biology. A discussion of potential challenges and future options for MMNP research is also included.

## Data Availability

Not applicable
